# Renal liposarcoma: case report and review of systemic treatment

**DOI:** 10.3332/ecancer.2021.1173

**Published:** 2021-01-14

**Authors:** Patricia Rioja, Guillermo Valencia, César Centurión-Rodriguez, Zaida Morante, Mercedes Bravo, Lourdes Huanca, Carlos Morante

**Affiliations:** 1 Medical Oncology Department, Instituto Nacional de Enfermedades Neoplásicas, Lima 15038, Peru; 2 Pathology Department, Instituto Nacional de Enfermedades Neoplásicas, Lima 15038, Peru; 3 Surgical Oncology Department, Instituto Nacional de Enfermedades Neoplásicas, Lima 15038, Peru

**Keywords:** liposarcoma, kidney neoplasms, nephrectomy

## Abstract

Liposarcomas are malignant mesenchymal tumours usually located in the retroperitoneum, rarely occurring as a single lesion in the kidney. We present a case of a 59-year-old male patient with a left renal mass detected by computed tomography scan. He underwent radical nephrectomy and the histopathological study reported a primary undifferentiated liposarcoma of the kidney without nodal involvement. After 15 months of surgery, he remained asymptomatic and without evidence of disease recurrence. The objective of this report is to present a case and literature review with current evidence of treatment options and prognostic factors for survival.

## Introduction

Liposarcomas are malignant neoplasms of mesenchymal origin that belong to the group of soft tissue sarcomas, constitute less than 1% of all malignant tumours and have a high infiltrative and metastatic potential [1]. They occur most frequently in the extremities (52%) and in the retroperitoneum (19%). The treatment of choice is complete surgical resection. The 5-year survival rate for a histologically well-differentiated liposarcoma is 83%, while for an undifferentiated one, it is about 20% [2]. In contrast, the prognosis of renal liposarcoma is poor, with a 5-year survival of 29%–39% after surgery [3].

Renal liposarcoma is an extremely rare neoplasm (1%–2% of primary renal sarcomas). It belongs to the group of sarcomas of the genitourinary tract, of which leiomyosarcomas are the most common, followed by liposarcomas. Liposarcoma of the kidney tends to be diagnosed incidentally in patients who present with renal mass, thoraco-lumbar pain and dyspnoea. Radical nephrectomy is the treatment of choice [4].

## Aim

The aim of this article is to report a rare case of a patient who was diagnosed as having undifferentiated renal liposarcoma based upon pathology examination of his radical nephrectomy specimen, and to provide an updated review of the literature description of renal liposarcoma, its treatment according to scenarios (perioperative, postoperative or advanced disease) and prognostic factors that have been documented to be related to survival of patients.

## Case report

A 59-year-old male patient, from Peru, with no relevant medical history, presented in September 2019 with a history of back pain, weight loss of about 5 kg, early feeling of fullness and night sweating of 8 months of evolution. His physical examination revealed a mobile mass without defined margins, located 12 cm from his left ribcage. The results of his routine haematology and biochemistry laboratory blood tests revealed moderate anaemia with a haemoglobin of 9.0 g/L and hyperkalaemia with a serum potassium of 5.8 mmol/L.

**Figure 1.**
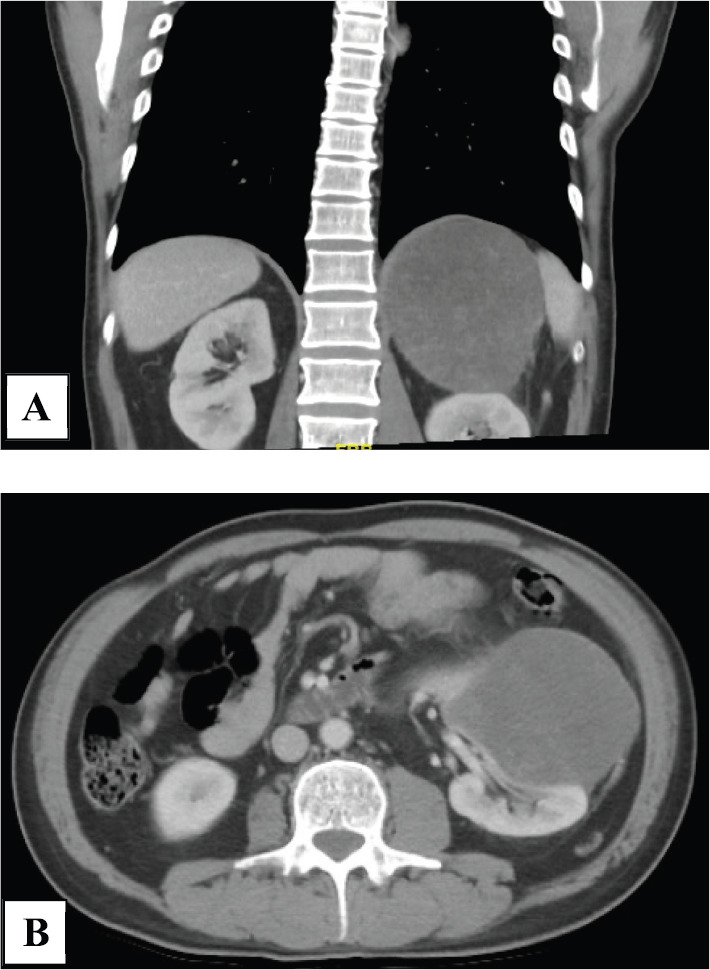
Computed tomography scan. (a): Abdominal CT scan showing a neoplastic lesion in the left kidney of heterogeneous density and (b): with alteration of the adjacent fat planes in its caudal pole.

He had computed tomography (CT) scan of abdomen which showed a left renal mass of heterogeneous density of 19 × 12 × 9 cm, which had defined borders and contours and which was not associated with any nodal or vascular involvement (Figure 1). Kidney cancer presumptive diagnosis was considered.

In October 2019, he underwent a surgery (left radical nephrectomy plus para-aortic lymphadenectomy), and during the procedure he was noted to have a left kidney dependent tumour that measured 20 × 19 cm with associated hilar and para-aortic lymph nodes.

**Figure 2.**
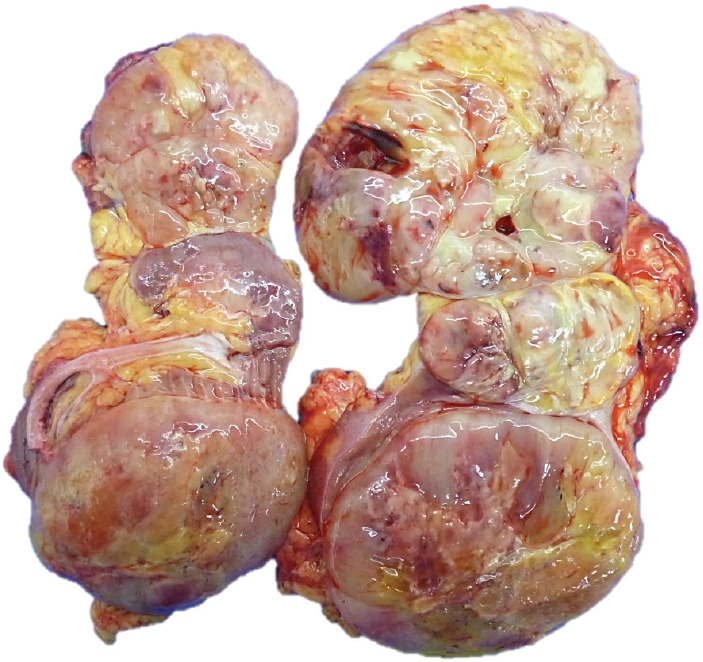
Macroscopy. Left kidney with the presence of a 23.0 × 12.0 × 10.0 cm tumour, solid in appearance, soft consistency, light brown in colour with necrotic areas in 30%, myxoid areas in 30% and haemorrhagic areas in 10%, which infiltrates sinus, renal capsule and perineal fat.

**Figure 3.**
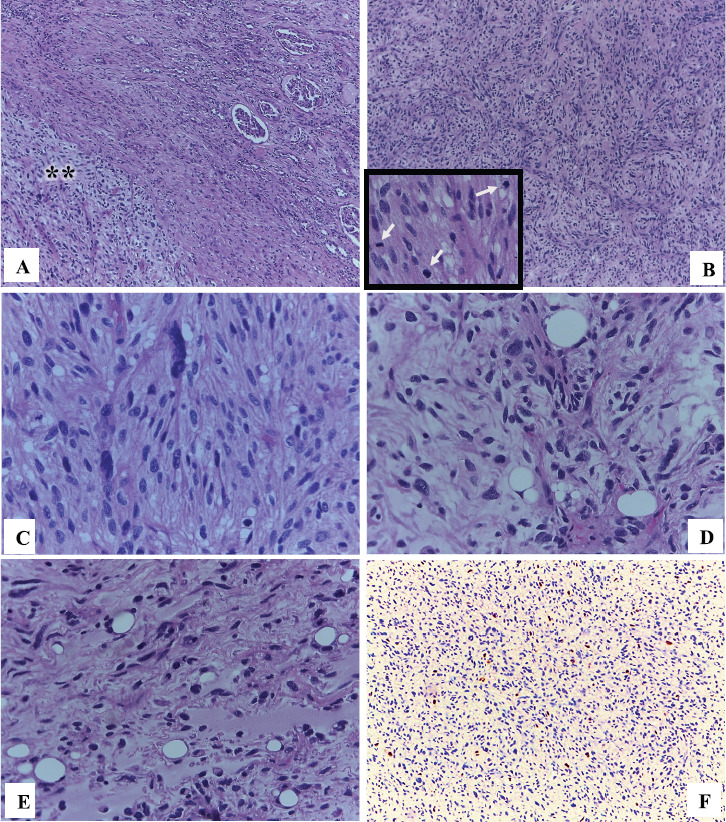
Microscopy. (a): In the upper right normal renal parenchyma with some glomeruli and towards the lower left a spindle cell proliferation (asterisks) (HE 50×). (b): This tumour proliferation is characterised by being formed by nests or fascicles of spindle cells with areas of greater cellular atypia and with high mitotic activity (arrows) (HE 100×). (c): Some cells present a higher degree of pleomorphism and hyperchromasia (HE 400×). (d) and (e): The areas of abrupt and focal transition from well-differentiated adipocytes to dedifferentiated tumour cells (HE 400×) were also identified. (f): Nuclear immunoexpression of MDM2 in tumour cells confirmed the diagnosis of dedifferentiated liposarcoma (IHC 100×).

Macroscopy pathology examination of the specimen was reported to have identified a left renal tumour that measured 23 × 12 × 10 cm which was noted to have a solid component, and which was soft in consistency, and which was light brown in colour and which contained areas of myxoid and haemorrhagic necrosis of 30% and 10%, respectively (Figure 2). Microscopy pathology examination of the specimen detected a cell proliferation of tumour cells that were arranged in a storiform pattern which infiltrated the renal sinus, capsule and the perirenal fat, with pleomorphic nuclei and high mitotic index (Figure 3). These findings were reported to be consistent with an undifferentiated liposarcoma of high histological grade, with 35 mitoses per ten high-power field (35/10 high-power field mitoses) and 30% of necrosis.

There was no evidence of metastatic spread to the Gerota’s fascia, renal pelvis, adrenal glands as well as there was no evidence of lymphovascular invasion by the tumour.

Immunohistochemistry staining study of the tumour with MDM2 showed that the tumour had exhibited positive nuclear immunostaining expression within the neoplastic cells (Figure 3), which did confirm the proposed diagnosis. A definitive final diagnosis of renal liposarcoma of the left kidney was made and this tumour was classified as clinical stage IIIB (T4N0M0 G3) for he had undergone radical surgical excision (radical nephrectomy and lymph node excision) as a primary treatment with negative surgical resection margins (R0). The patient has been undergoing regular follow-up assessments with clinical examination, laboratory tests and radiology imaging assessments.

After 15 months of follow-up, the patient has remained well with no evidence of local recurrence or distant metastases based upon the results of his clinical examination, laboratory blood tests and CT scan images. He will continue to have regular follow-up assessments.

## Discussion

Renal liposarcomas are extremely rare solitary neoplasms described in case reports. Literature reviews have reported 17 cases (14.3%) out of 119 reported cases of kidney sarcomas [5, 6]. Liposarcomas of the kidney are part of sarcomas of the genitourinary tract which represent 0.8% of all renal neoplasms and 2%–3% of malignant kidney tumours. Liposarcomas of the kidney have been reported in patients whose ages have ranged between 36 years and 86 years and liposarcomas of the kidney have been stated to be three times more frequently encountered in women in comparison with men [7, 8].

Most published cases of liposarcoma of the kidney had reported well differentiated tumours, with an average tumour size of 5 cm and the reported symptoms had included: pain, abdominal mass (most frequent), weight loss and haematuria (rare presentation) [10]. The most frequent haematogenous metastases have been reported in the lungs, lymph nodes and liver [3].

Radiology imaging studies that have been utilised for the evaluation of liposarcoma of the kidney do include: ultrasound scan of renal tract which does show hypo- or hyper-echoic lesion within the kidney, CT scan of kidney with contrast and magnetic resonance imaging scan of renal tract with contrast. Utilisation of pathology examination including microscopy examination of the kidney tumour supported by immunohistochemistry staining studies of the kidney tumour is the means by which a definitive diagnosis of primary liposarcoma of the kidney is definitely diagnosed.

There are five histological sub-types of liposarcoma of the kidney including: well-differentiated liposarcoma, myxoid liposarcoma, round cell liposarcoma, undifferentiated liposarcoma and pleomorphic liposarcoma. The myxoid type is the most frequent (60%), followed by the well-differentiated (25%), pleomorphic (10%), undifferentiated and round cells (<5%). Well-differentiated liposarcomas of the kidney have a high local recurrence rate and 15% rate of distant metastasis, with a 5-year mortality of 30%, whereas pleomorphic liposarcoma of the kidney, those of pure round cells and undifferentiated types of liposarcoma of the kidney tend to metastasise widely [5]. In the case of undifferentiated liposarcomas of the kidney, they tend to be non-lipogenic liposarcoma of varying histological grade that often occur in conjunction with a well-differentiated liposarcoma component. The amount and histological grade of the undifferentiated component does not generally show prognostic significance; however, in retroperitoneal tumours, high-grade morphology has been associated with decreased survival [19].

The immunohistochemistry studies of renal liposarcoma tend to show tumour cells that exhibit positive staining for murine double-minute type 2 (MDM2) and cyclin-dependent kinase 4 (CDK4) [11, 20]. In addition, they exhibit negative staining for pancytokeratin, EMA, desmin and HMB-45 and protein S100 [20, 21]. It has been stated that most renal liposarcomas usually tend to be derived from the renal capsule (most frequent site of presentation) or the renal sinus [12]. Primary tumours of the renal capsule are infrequent, and liposarcomas on this site are very uncommon. Ciccarello *et al* [13] reported 18 cases of liposarcoma with renal capsule involvement, including one case containing a fatty thrombus in the vena cava [14]. The histological type found in our case which was a case of undifferentiated liposarcoma of the kidney is also rare and there was no involvement of renal capsule noted by us.

The differential diagnoses of primary liposarcoma of the kidney include: angiomyolipomas (their main one since both are large lesions with fat content), dedifferentiated pleomorphic sarcoma, pleomorphic liposarcoma and myxoid liposarcoma, all of which lack the differentiated liposarcoma component and do not express MDM2. Another one is well-differentiated liposarcoma, however mitotic activity in these tumours is usually low or absent and histologically smooth with conspicuous lipoblasts, and also do not express MDM2 [9, 22].

The main management of primary liposarcoma of the kidney is radical nephrectomy. Few cases have reported the use of adjuvant treatment (chemotherapy or radiotherapy) in the management of primary liposarcoma of the kidney with discordant results. In general, an operated sarcoma with appropriate tumour-free resection margins (complete resection of tumour and any invaded organ with negative margins) has been the most common treatment option which is ensued by continuous and regular follow-up with clinical assessment, laboratory blood tests and radiology imaging at regular intervals. It has been reported that postoperative radiation therapy has no impact on recurrence rate [3, 12]. Surgical resection with free margins provides a high probability of cure, which differs from the poor prognosis in general for genitourinary tract sarcomas (with a 5-year survival rate of 29%–39%), due to the high proportion of patients who manifest with *de novo* metastatic disease and large tumour size at the time of their initial presentation [3, 7].

Chemotherapy is used in liposarcoma of kidney tumours that cannot be completely resected, in recurrent disease or in distant metastases. However, its benefit is minimal and not many studies have been undertaken. Perez *et al* [15] reported that chemotherapy agents such as doxorubicin and ifosfamide demonstrated activity on both retroperitoneal and truncal liposarcoma but have not demonstrated differences in survival (69 months median survival for truncal versus 78 months for retroperitoneal liposarcomas, *p* = 0.668). Livingston *et al* [16] performed a study in patients with retroperitoneal dedifferentiated liposarcoma (*n* = 82) which was divided in two groups: one receiving neoadjuvant chemotherapy for localised or locally advanced disease (*n* = 31) and another receiving chemotherapy for unresectable/metastatic or recurrent disease (*n* = 51). With regard to the result of the study, Livingston *et al* [16] reported the following: the median overall survival from the start of chemotherapy was 29 months (CI: 95%, 24–40). The response rates were assessed by CT scan and Response Evaluation Criteria in Solid Tumours (RECIST): partial response was found in 20% of the cases, stable disease was found in 40% of cases and disease progression was found in 40% of cases. Most patients received anthracycline-based combination chemotherapy (88%), with doxorubicin and ifosfamide being the most common first-line regimen, with a median of four cycles in all regimens. Chemotherapy had a response rate of 24% and a clinical benefit rate of 44%. In addition, 50% of patients received second-line chemotherapy (most often with gemcitabine/docetaxel at 56%) and 12% received third line. The median survival from diagnosis was 45 months (95% CI, 36–66 months) [16].

It has been iterated that the most important prognostic factors for this pathology include: the degree of differentiation, the tumour size, the histological type, the anatomical location of the tumour and completeness of resection of the tumour [17, 18]. The reported local recurrence rates are between 20% and 85% [15]. Wang *et al* [14] documented 1-year survival rate of 86%, 3-year survival rate of 41% and 5-year survival rate of 15%, with a median survival of 28 months but 8 to 10 months in recurrence or metastatic disease. Recurrences have been reported up to 13 years after initial surgery [5].

## Conclusions

Renal liposarcomas are extremely rare tumours that develop from mesenchymal tissue. Their asymptomatic onset does limit the diagnosis of primary liposarcoma of the kidney and the manifestations of the disease tend to be non-specific symptoms. The treatment of choice of primary liposarcoma of the kidney is radical nephrectomy. Chemotherapy and radiotherapy have not shown survival benefit in neither the neoadjuvant nor the adjuvant setting. Prognostic factors of primary liposarcoma of the kidney are related to the tumour characteristics and the type of surgery that has been undertaken.

Because primary liposarcoma of the kidney is an extremely rare disease, data related to the tumour is limited; therefore, it is very important to encourage prospective and randomised clinical trials that are focused on treatment of the tumour.

## Conflicts of interest

The authors have no conflicts of interest to declare.

## Funding statement

No funding was received for this work.

## Authors’ contributions

P Rioja, G Valencia and Centurion-Rodriguez were involved in manuscript preparation, reference hunting and final editing. Z Morante was involved in reference hunting and final editing. Dr Bravo M and Huanca L were concerned with the histopathologic and immunohistochemical diagnosis. Dr C Morante was involved in final editing.
